# Deformation characteristics and mechanism of Mala paleo-landslide during the Miaowei reservoir initial impoundment period

**DOI:** 10.1038/s41598-024-55559-8

**Published:** 2024-03-01

**Authors:** Zhijie Mai, Xi Hu, Lianke Li, Jiacheng Hou

**Affiliations:** Badong National Observation and Research Station of Geohazards, China University of Geosciences, Wuhan, 430074 China

**Keywords:** Mala landslide, Reservoir water level rise, Buoyancy weight-reducing landslide, Retrogressive type landslide, Civil engineering, Engineering

## Abstract

This study employs a multifaceted approach, encompassing field investigations, borehole surveys, surface deformation displacement monitoring, deep-seated deformation monitoring, and numerical simulation analysis, to conduct an exhaustive examination of the deformation processes and characteristics exhibited by the Mala Landslide. The findings elucidate a close correlation between the deformation of the Mala Landslide and the elevation of the reservoir water level. During the escalation of the reservoir water level, the landslide body progressively developed surface cracks, spanning from the frontal edge to the rear edge. The centre of the sliding body is situated in the central-lower downstream region, and presently, the landslide is undergoing a phase of comprehensive creep deformation. Due to the reservoir water level fluctuation rate being greater than the permeability coefficient, the deformation of the landslide displays a delayed response. As the reservoir water level reaches 1401 m during high-water operation, the two important ingredients, the buoyancy weight reduction effect and the influence of submerged reservoir water, significantly reduce the sliding resistance of the sliding mass, thereby exacerbating the deformation of the landslide. Following a comprehensive analysis of the findings, it can be firmly concluded that this landslide conforms to the characteristic traits of a typical buoyant force reduction type-retrogressive type landslide.

## Introduction

Large-scale hydraulic engineering projects often encompass reservoir areas that host various ancient landslides of differing sizes, which can undergo reactivation during the impoundment and operational phases^1,2^. Notable examples include the Vaiont landslide in Italy^3^, the Canelles landslide in Spain^4^, and the Qianjiangping landslide in China^5,6^, all of which have resulted in substantial human and financial losses. The Miaowei Hydropower Station is situated in the upper reaches of the Lancang River, near Jiuzhou Town in Yunlong County, Yunnan Province. It is the lowest downstream cascade station within the framework of the seven cascade development plans for the upper Lancang River. The station boasts an installed capacity of 1400 MW and achieves an average annual power generation of 65.56 × 10^8^Kw h^7,8^. Preliminary investigations conducted prior to the impoundment at the Miaowei Hydropower Station revealed the existence of several ancient landslides within the reservoir area. The fluctuation of reservoir water levels during operation poses the potential risk of reactivating and inducing sliding damage in some of these ancient landslides^9^.

The Mala Landslide is situated between the Wayout River and the Amara River, both right bank tributaries of the Lancang River. It is a typical reactivated ancient landslide induced by the impoundment of the Miaowei Hydropower Station. The reservoir water level at the Miaowei, and then slowly rose by 1401 m. In April 2017, surface displacement monitoring points detected signs of slope deformation. By mid to late August, the deformation measurements exhibited a significant increase, and subsequently, deformation continued to advance without abating. The instability of this landslide has the potential to cause significant loss of life and property, prompting widespread concern from the local government and community.

Before impoundment, the engineering geological survey and geological drilling were conducted on the Mala landslide to understand its characteristics. In order to mitigate the landslide risk, ongoing monitoring was carried out during the impoundment period, which included surface deformation displacement monitoring, deep-seated deformation displacement monitoring, and water level observations. Based on the aforementioned work, this paper analyzes the process and characteristics of the reservoir-induced deformation of the Mala landslide. Furthermore, it combines numerical simulation method to analyze the mechanism of reservoir-induced acceleration deformation and failure of the Mala landslide, aiming to provide theoretical basis and data support for relevant hydraulic and hydropower reservoir landslide prevention and control projects.

## Materials

### General description of the landslide

**Figure 1:**
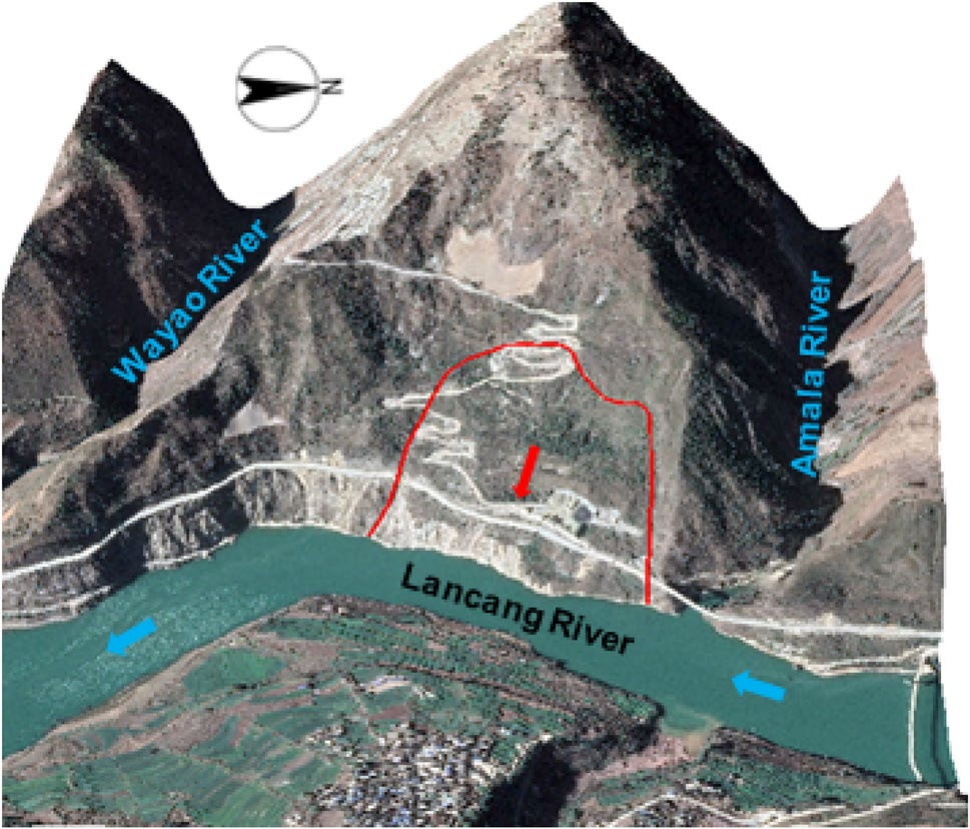
Mala paleo-landslide.

As shown in Fig. 1, the upstream of the Mala landslide is the Amara River, and the downstream is the Wayao River. The elevation of the landslide area ranges from 1380 to 1560 m, extending approximately 420 m in the downstream direction. The thickness of the sliding body varies from 10 to 75 m, with a total volume of approximately 4.3 × 10^6^ m^3^. Below an elevation of 1440 m, the terrain becomes steeper, with slopes ranging from 40° to 60°. In the section of the landslide with elevations ranging from 1440 to 1650 m, the terrain is relatively gentle, and the slope typically ranges from 20° to 35°. On the right bank of the reservoir, there are two roads passing through the lower and middle parts of the landslide.

The slope where the landslide is located has a strike of N20° E, while the rock occurrence is N0°–10° SW∠60°–80°, the direction of the strike of the landslide intersects with the rock stratum at a small angle, and the superficial rock mass exhibits strongly toppling deformation. From upstream to downstream direction, the angle between the slope strike and the rock stratum gradually increases, and the toppling deformation weakens progressively in the downstream direction. Additionally, in the lower part of the sliding mass, there is also a distribution of a certain thickness of toppling deformation rock mass, with the bottom boundary of the toppling deformation rock mass located near an elevation of 1380 m.

The primary type of groundwater within the landslide body is bedrock fissure water. It is primarily replenished by atmospheric precipitation and discharges into the Lancang River valley. Geological exploration boreholes conducted prior to the impoundment revealed that the groundwater in the bank slopes is buried relatively deep and is generally at the same level as the river water in the nearby valley segment.

### Material composition and structure characteristics

**Figure 2:**
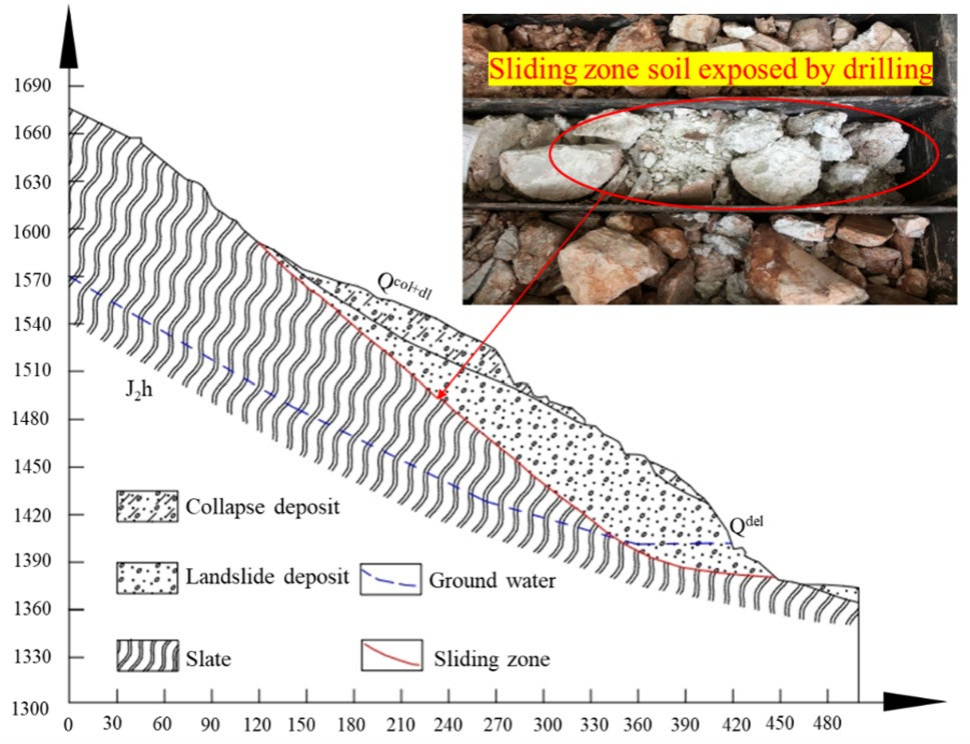
Material composition and structure characteristics.

Based on on-site investigations and drilling surveys, the sliding mass is primarily composed of the collapse deposit (Q^col+dl^) and landslide deposit (Q^del^). The collapse deposit containing a gravel silty clay layer is primarily distributed in the surface layer of the sliding mass, with a thickness ranging from 4 to 20 m. The debris from the landslide deposit is mainly located in the middle to lower parts of the sliding mass and constitutes a major portion of it, with a thickness ranging from 40 to 70 m. The longitudinal cross-sectional deposit of the landslide exhibits a "crescent" shape, characterized by a thinner deposit layer at the rear edge and thicker deposits in the central and front portions (Fig. 2).

The location of the sliding zone closely aligns with the bedrock-cover boundary, and the sliding zone consists mainly of the lower part of the covering layer and the strongly weathered top of the bedrock. Within the sliding zone, the drill core samples of the rock (or soil) exhibit fragmented and brecciated characteristics, consisting primarily of rock fragments with a small amount of silty clay. It is inferred that the sliding zone material originates from the debris layer of the landslide deposit and the strongly weathered bedrock. Based on a comprehensive analysis of core samples extracted from boreholes, it is estimated that the thickness of the sliding zone ranges from 1 to 4 m, with a maximum depth of 70 m.

The bedrock of the sliding bed is the Jurassic Series Huaikou Formation (J_2_h) shale, which forms steeply dipping, moderately thin-layered rock units. The rock is fragmented with well-developed joint fractures. And the rock mass exhibits strongly toppling deformation, with the toppling deformation rock stratum ranging from strongly weathered to weakly weathered. The strongly weathered layer has a thickness of 5–15 m, while the weakly weathered layer ranges from 20 to 80 m in thickness. The rock cores extracted from the survey boreholes transition from fragmentary to laminar, short columnar, and columnar as depth increases, with the integrity of the rock gradually improving and the degree of weathering diminishing.

## Deformation characteristics

### Ground deformation feature

**Figure 3:**
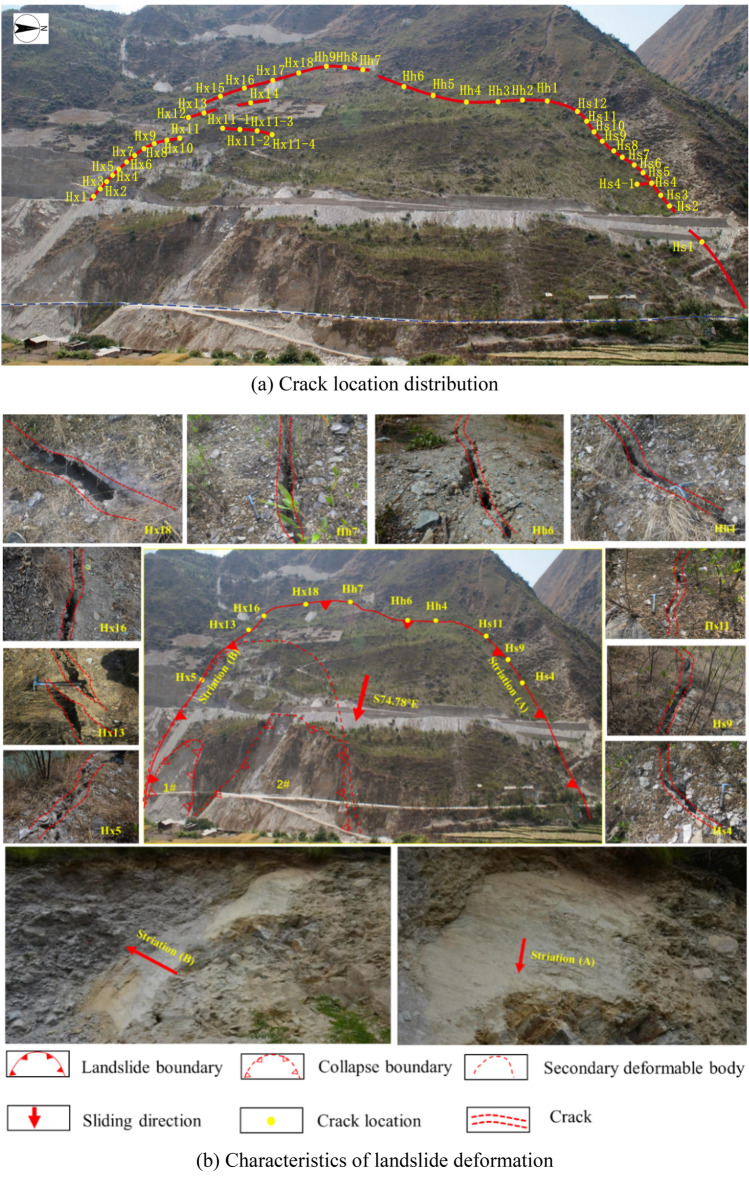
Ground deformation feature.

Since the impoundment began on April 21, 2017, the water level in the reservoir rose from an elevation of 1372.7–1392.8 m by June 2017. As a result, the front edge slope of the sliding mass (near the downstream side) has caused a certain range of bank collapse (Fig. 3b 1# collapse). In the mid to late July of 2017, the reservoir water level stabilized at an elevation of around 1401 m, several small-scale collapses occurred at the front edge (Fig. 3b 2# collapse). Surface cracks began to appear successively from the front edge to the rear edge of the sliding mass, with the development of surface cracks primarily concentrated along the perimeter of the ancient landslide. In December 2017, the cracks at the front edge had partially connected, extending for approximately 100 m with an opening width of about 5–15 cm. By March 2018, a total of 42 cracks had been identified during on-site investigations. Except for Hs3 and Hx11, which were shear cracks, all others were tension cracks. The field survey results indicated that the average opening width of these slope cracks ranged from 10 to 20 cm, reaching up to 30 centim in some local areas. Essentially, the perimeter cracks within the sliding mass were interconnected. Please refer to Fig. 3 for the on-site survey results of cracks within the landslide area.

Taking into consideration Fig. 3, we will proceed to analyze the average width, extension direction, and characteristics of the sliding mass within the landslide area through investigations of cracks both upstream, at the rear, and downstream of the sliding mass. The details are as follows:

1. Upstream

The trace lines of the upstream cracks (Hs1–Hs12) closely align with the landslide boundary. Among these, cracks Hs4, Hs6, Hs9, and Hs11 were the first to appear on the upstream side of the landslide boundary, with an average width exceeding 10 cm. Other cracks are distributed on both sides of these four cracks, indicating that the formation of the other cracks occurred as a result of increased deformation under the gravitational pull of the sliding mass. Furthermore, the overall trend of the cracks extends from Hs11 towards Hs4, suggesting that the deformation direction of the upstream rock (or soil) mass leans towards the downstream direction.

2. Rear

The number of rear cracks (Hh1–Hh9) is relatively smaller, but the cracks tend to be wider and longer in extension. Cracks Hh1–Hh6 intersect with the upstream cracks, and Hh7–Hh9 intersect with the downstream cracks. Among these, cracks Hh1 and Hh3–Hh6 formed earlier than Hh2, with an average width exceeding 10 cm. The overall trend of these cracks extends from Hs6 towards Hs1, which is similar to the deformation trend of the upstream cracks. The average width of cracks Hh7–Hh9 varies, and the width of these cracks gradually decreases from Hh7 to Hh9, indicating that the extension direction of the cracks is from Hh7 to Hh9.

3. Downstream

The downstream cracks (Hx1–Hx5 and Hx15–Hx18) are in proximity to the landslide boundary, while the remaining cracks deviate from it. The cracks in the Hx1–Hx5 and Hx15–Hx18 formed relatively early, with an average width exceeding 10 cm. Crack Hx11 tends to intersect with Hx11–4 and shows a trend of extending towards the central part of the landslide. This is due to relative displacement occurring within the landslide during the impoundment process, leading to the formation of a secondary sliding mass (Fig. 3b). Based on the location of crack Hx11, it can be concluded that the sliding direction of the downstream mass tends to downstream. Additionally, the bedrock on the south side of the downstream landslide boundary partially obstructs the downward movement of the rock and soil mass. Therefore, overall, the deviation angle from the upstream rock (or soil) mass is relatively smaller.

Furthermore, by excavating trenches both upstream and downstream of the sliding mass to expose the ancient sliding surface, distinct scratch marks were observed. The occurrence of the sliding surface is N70° E, NW∠60°. The upper portion of the sliding surface consists of gravelly soil, while the lower portion comprises strongly toppling deformation rock formations with the occurrence of the rock strata is N5° E, NW∠30–40°.

### Surface displacement response

**Figure 4:**
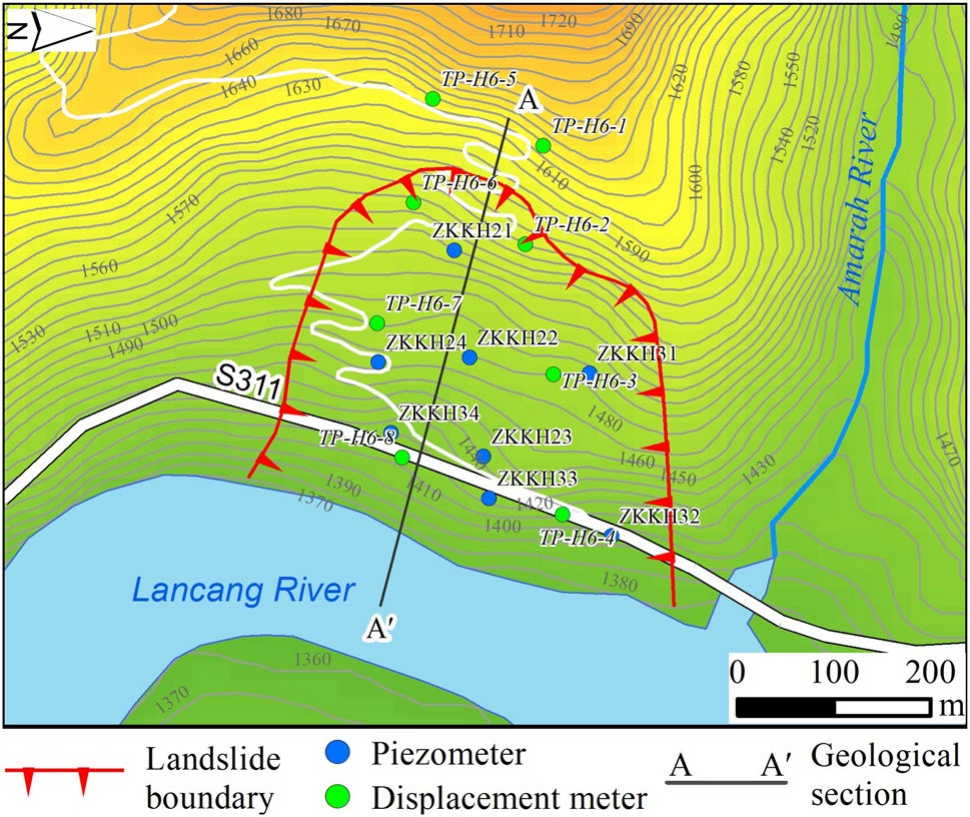
Layout of landslide deformation displacement monitoring points.

**Figure 5:**
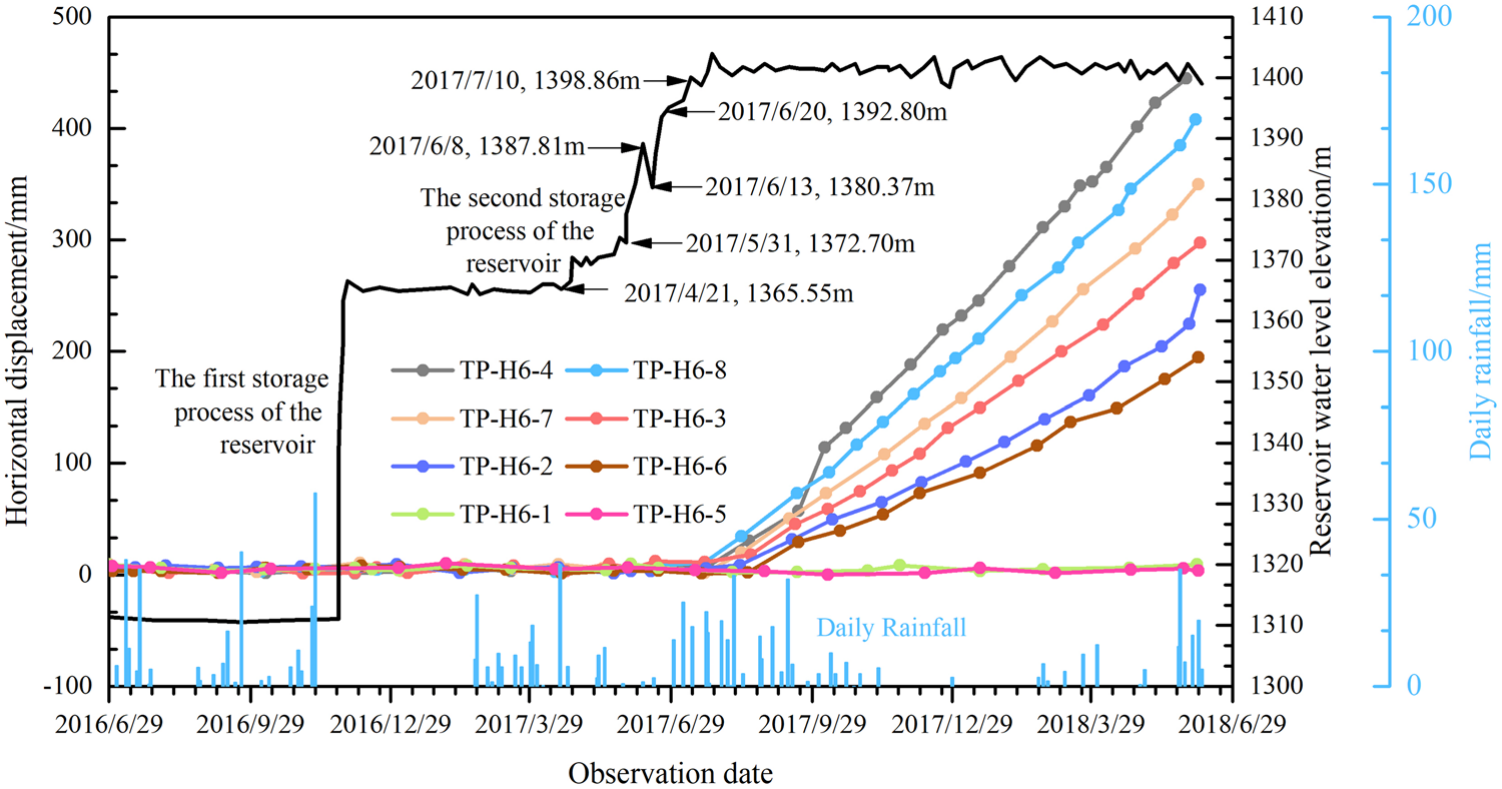
Deformation curve of Mala landslide surface displacement monitoring.

Surface deformation displacement monitoring involves the installation of eight GNSS monitoring devices within and around the landslide area, numbered TP-H6-1 to TP-H6-8 (see Fig. 4). These GNSS monitoring devices started acquiring initial observations on June 29, 2016, and the horizontal displacement curves for each monitoring point are depicted in Fig. 5. It can be observed that after the impoundment, deformation occurred at 6 monitoring points within the perimeter of the landslide (TP-H6-2, TP-H6-3, TP-H6-4, TP-H6-6, TP-H6-7, TP-H6-8), while two monitoring points outside the landslide perimeter (TP-H6-1, TP-H6-5) did not exhibit deformation.

During the initial impoundment (from June 29, 2016 to March 29, 2017), as the water level remained below the sliding surface, the water level fluctuations had minimal impact on the sliding mass. The displacement measurements at each monitoring point were very small, and deformation was nearly absent. However, by April 2017, as the reservoir water level gradually rose, it exceeded the sliding surface at the base of the landslide. Consequently, deformations began to emerge at each monitoring point. The deformation curves at each monitoring point generally exhibited an exponential pattern. From mid-June 2017 to mid-August 2017, the deformation rates were relatively slow. Starting from mid-August 2017, the deformation at each monitoring point accelerated, and later, the deformation rates became relatively stable, approaching uniform deformation (Fig. 5).

From June 8, 2017, to June 13, 2017, there was a sudden drop in the reservoir water level, and there were no significant changes in the deformation curves at various monitoring points. Additionally, the deformation curves at these monitoring points did not exhibit any significant abrupt changes due to rainfall (Fig. 5). This suggests that the sudden rise in water level is an important factor in triggering the formation of Mala landslide after excluding the two factors of falling water levels and rainfall. Furthermore, according to Fig. 5, there was no significant change in surface deformation displacement during the impoundment process (from April 21, 2017 to July 15, 2017). Instead, after the reservoir water level stabilized at 1401 m, there was a noticeable increase in surface deformation displacement (after July 15, 2017). This indicates that the deformation of the landslide exhibits a certain degree of hysteresis.

Furthermore, the deformation conditions at various monitoring points exhibit synchronicity, with deformation occurring almost simultaneously. Moreover, the deformation trends are consistent, with deformation magnitudes showing the following pattern: landslide front (TP-H6-4, TP-H6-8) > central part of the landslide body (TP-H6-7, TP-H6-3) > landslide rear (TP-H6-2, TP-H6-6). This aligns with the results obtained from surface surveys.

### Depth of displacement with inclinometer

**Figure 6:**
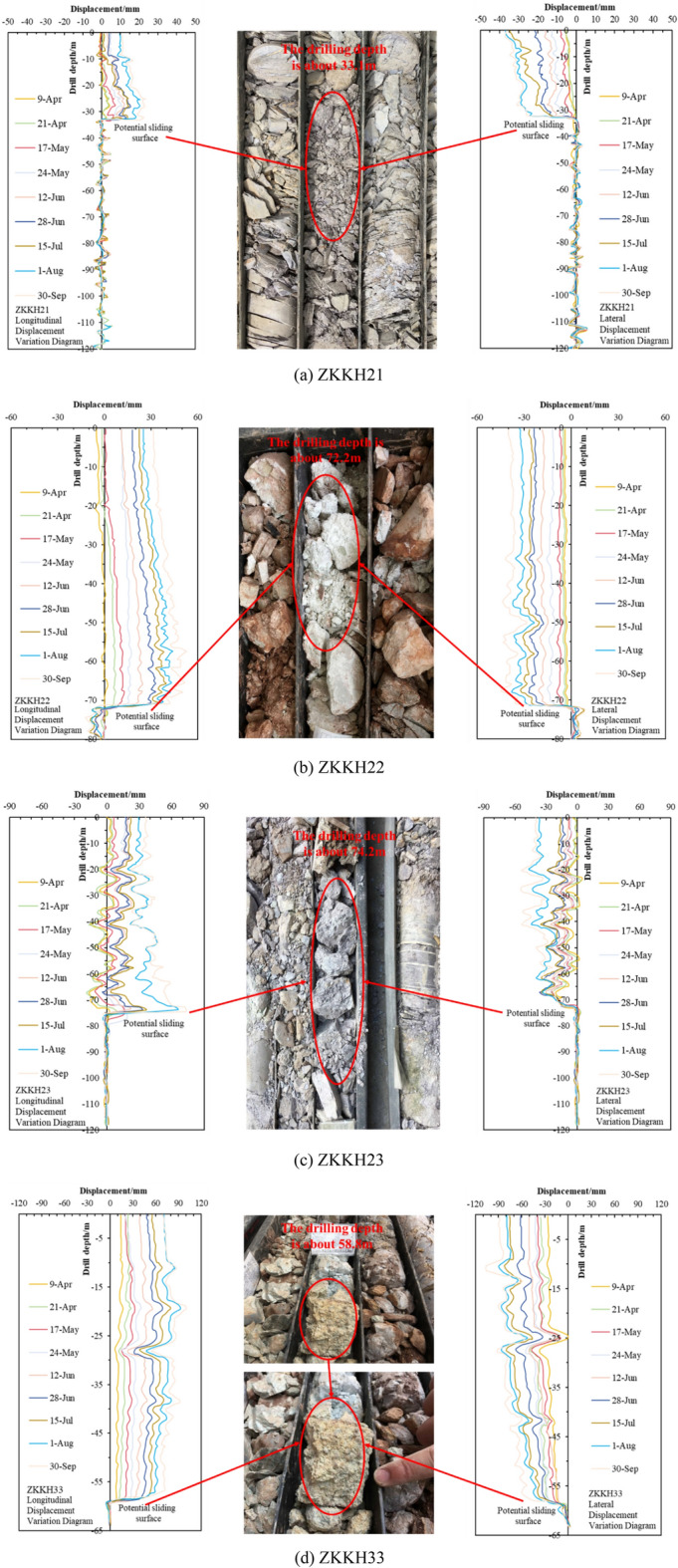
Deep displacement variation and slip zone detail diagram.

The deep-seated displacement monitoring includes a total of eight boreholes with the following designations: ZKKH21–ZKKH24, ZKKH31–ZKKH34, as illustrated in Fig. 6. Eliminating the influence of bending deformations in the inclinometer pipes themselves, the data from each monitoring point exhibit similar trends. To gain a more detailed understanding of displacement variations in the rear, central, and front sections of the landslide, the profile composed of ZKH21–ZKH22–ZKH23–ZKH33 was chosen to conduct an analysis of the deep-seated displacement characteristics within the landslide body.

In the displacement curve for ZKKH21, the deformation near the bottom of the borehole exhibits minimal displacement. However, there is a significant displacement surge between depths of 32.5 m and 33.4 m and the changed curves are continuous and flat, which aligns with the characteristics of shear displacement. And according to the core from the ZKKH21 borehole, at a depth of 33.1 m, there is a weak interlayer (Fig. 6a), it is estimated that the depth of the sliding surface is approximately at a depth of 33.1 m.

In the displacement curves for ZKKH22 and ZKKH23, the monitoring curves at depths of 72.0 m and 74.0 m, respectively, exhibit characteristics consistent with shear displacement. The core samples from the ZKKH22 and ZKKH23 boreholes reveal that, at depths of 72.2 m and 74.2 m, the cores are fragmented and show significant weathering, with the gravel displaying a notable degree of rounding (Fig. 6b,c). Combining this information, it is inferred that the slip surface is located at a depth of 72.2 m (ZKKH22) and 74.2 m (ZKKH23).

In the displacement curve for ZKKH33, there is a noticeable surge in displacement at a depth of 58.0 m, and below this depth, there are no significant indications of deformation. Moreover, based on the core samples obtained from borehole ZKKH33, at a depth of 58.8 m, the core samples exhibit strongly weathering (Fig. 6d), closely resembling the exposed sliding surface at the upstream landslide shear outlet. Combining the information, it can be inferred that the location at a depth of 58.8 m corresponds to the sliding surface.

**Figure 7:**
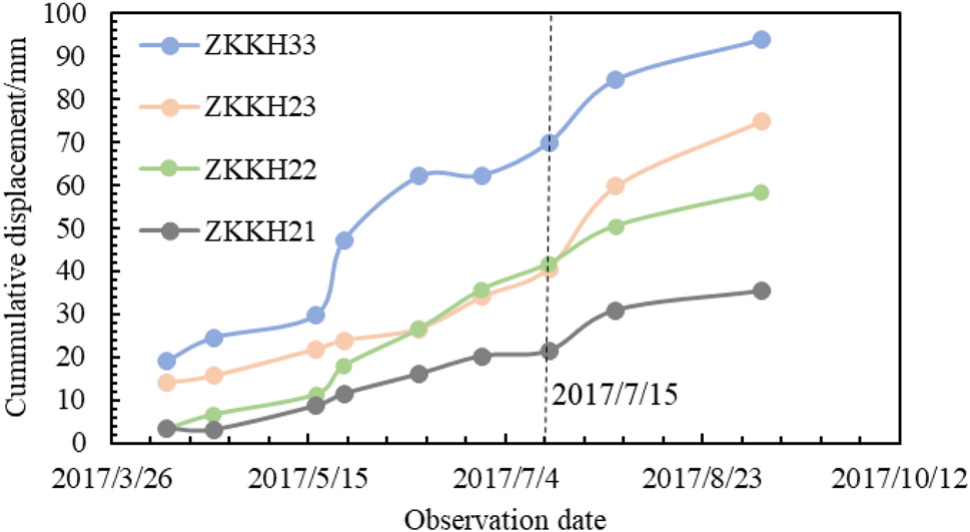
Deep cumulative displacement.

In deep-seated displacements, the monitoring displacement curves of individual point exhibit a generally consistent trend, indicating that the mass above the sliding surface is experiencing overall sliding and is in a state of overall creep deformation. Furthermore, the cumulative monitoring displacement data for ZKKH21, ZKKH22, ZKKH23, and ZKKH33, as shown in Fig. 7, follows a trend consistent with the surface deformation. The displacement is more pronounced at the landslide front (ZKKH33), followed by the central portion of the landslide (ZKKH22 and ZKKH23), and lastly, the rear part of the landslide (ZKKH21). This indicates that the Mala landslide is a retrogressive type landslide, aligning well with the deformation characteristics observed during the ground deformation survey. It is worth noting that since July 15, 2017, there has been a noticeable increase in deep cumulative displacement (Fig. 5). This indicates that, following the stabilization of the reservoir water level at around 1401 m, the groundwater level is gradually stabilizing. The change of groundwater level is an important factor in the deformation and displacement of the landslide.

## Numerical simulation

### Model settings and param

Based on the monitoring data, it is evident that the impoundment in the reservoir is the primary factor influencing the deformation and instability of the landslide. This study will utilize a numerical simulation method to precisely evaluate the impact of this factor on the stability of the landslide. Geological survey data from the Mala landslide will be employed to create a two-dimensional model, with the A–A′ profile (Fig. 4). The GEO-STUDIO numerical simulation software will be used to analyze the seepage field and stability of the landslide. The model will be constructed based on the actual topography and geological layers. And the physical–mechanical param for the debris clay layer, landslide deposits, sliding layer, and bedrock will be determined through physical mechanics experiments. Specific calculation param are detailed in Table 1.

**Table 1:** Physical and mechanical parameters of rock and soil layers.

Classification of soils and rocks	Φ (°)	C (kPa)	γ (kN/m^3^)	Φ′ (°)	C’ (kPa)	γ_sat_ (kN/m^3^)	E (MPa)	μ	Tensile strength (kPa)	Permeability coefficient
	Natural state			Saturation state						
Collapse deposit	28.50	26.00	22.00	26.30	24.80	22.40	710.00	0.25	0	1.00 × 10^–5^
Landslide deposit	30.50	25.00	21.50	27.20	23.50	21.90	790.00	0.25	0	5.00 × 10^–5^
Sliding Zone	25.00	8.00	17.00	21.00	6.00	19.00	620.00	0.23	0	1.34 × 10^–3^
Weathered bedrock	36.00	320.00	24.00	33.00	280.00	24.60	840.00	0.21	1200.00	–

**Figure 8:**
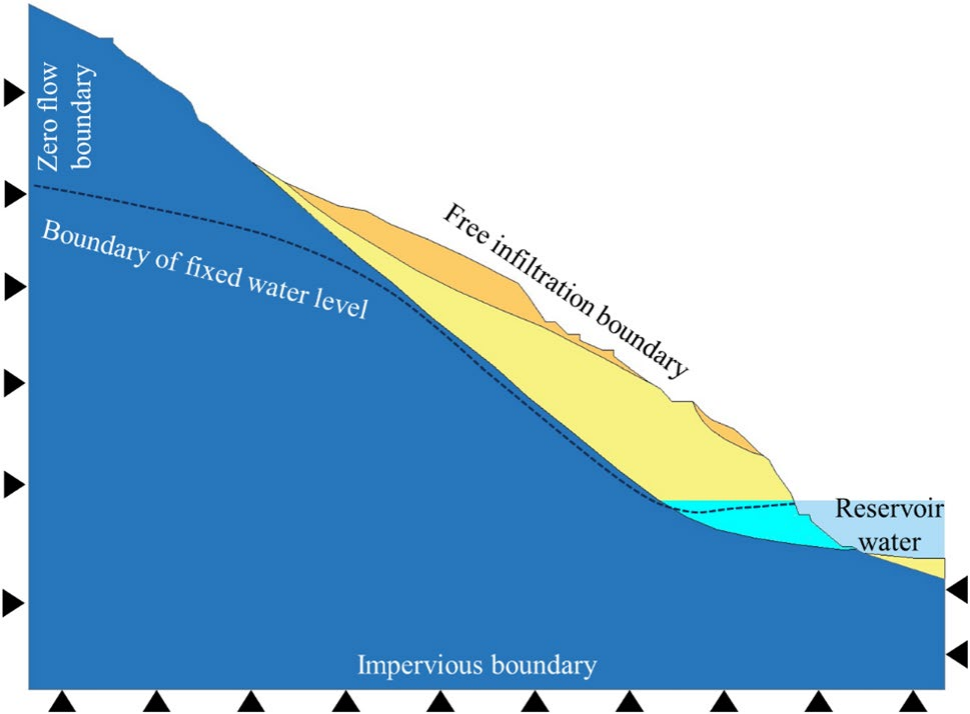
Schematic diagram of boundary setting.

The boundary conditions for the model are determined based on hydrogeological considerations (Fig. 8). It is assumed that both the sliding mass and bedrock are composed of homogeneous materials. Fixed constraints are applied at the bottom, while normal constraints are applied at the left and right ends. The upper surface is treated as a free infiltration boundary, and the lower surface is considered impermeable. Zero flow boundaries are applied on both sides above the water level, and given head boundaries are established below the water level, set at the water head at each respective location. Specifically, the water head on the left boundary is set to the initial groundwater level, and on the right boundary, there's a variable head boundary due to changes in the reservoir water level. Calculations are based on the natural state of the slope before reservoir filling, with the reservoir water level during the filling period applied as a boundary condition on the slope surface.

Based on the monitoring data, considering the period from May 31, 2017, to July 10, 2017, which spans 41 days, with an average daily water level rise rate of approximately 0.7 m per day, the relationship between the water level elevation H(t) and time t is as follows:

$$H\left(t\right)=\left\{\begin{array}{l}1372.70+0.7t, \quad0\le t\le 41\\ 1401, \quad\quad\quad\quad\quad\,\, t>41\end{array}\right.$$

### Seepage field and stability

**Figure 9:**
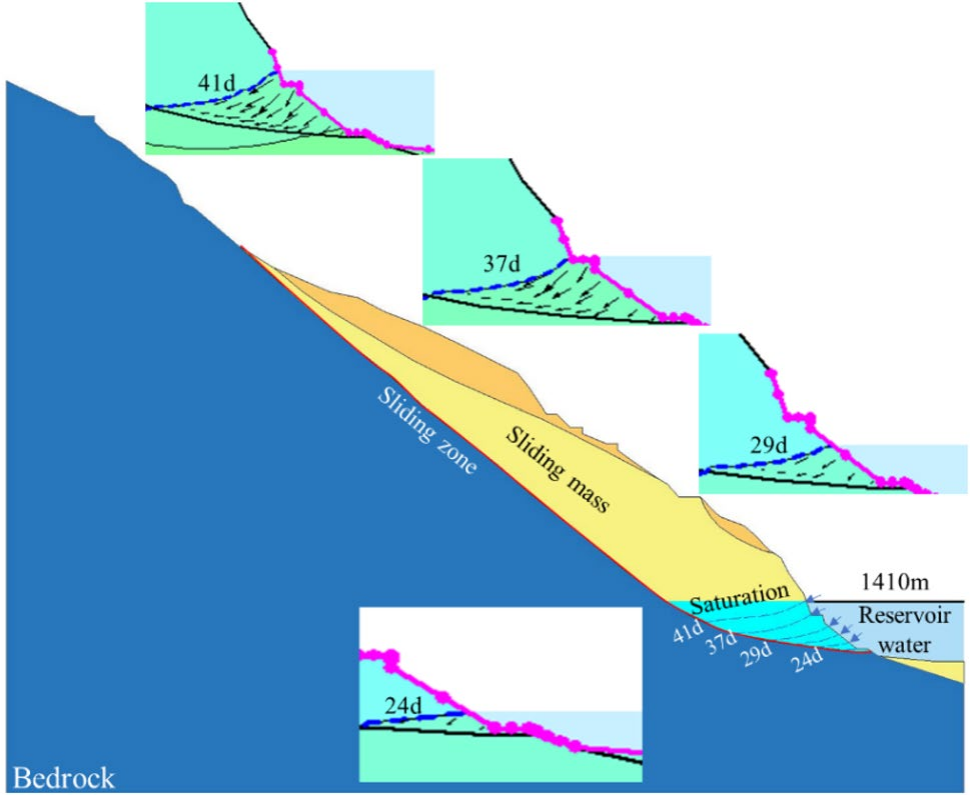
The variation of infiltration line with the increase of reservoir water level.

According to the analysis of numerical simulation results, it is observed that as the reservoir water level rises, the infiltration line within the slope ascends, and the infiltration line exhibits a concave shape towards the interior of the slope (Fig. 9). During the rising of the reservoir water level, the water level within the slope lags behind the reservoir water level, resulting in a water level difference. Water infiltrates into the slope, generating dynamic water infiltration pressure and pore static water pressure directed towards the interior of the slope. Therefore, it can be concluded that during the reservoir level increase, the mechanical behavior of the Mala landslide involves a combination of the infiltration force generated by dynamic water pressure and the buoyant force produced by pore static water pressure. However, due to the influence of the sliding surface morphology (featuring an anti-slide segment), the buoyancy-induced reduction in weight is more significant than the underground water infiltration pressure, making buoyancy the primary factor.

**Figure 10:**
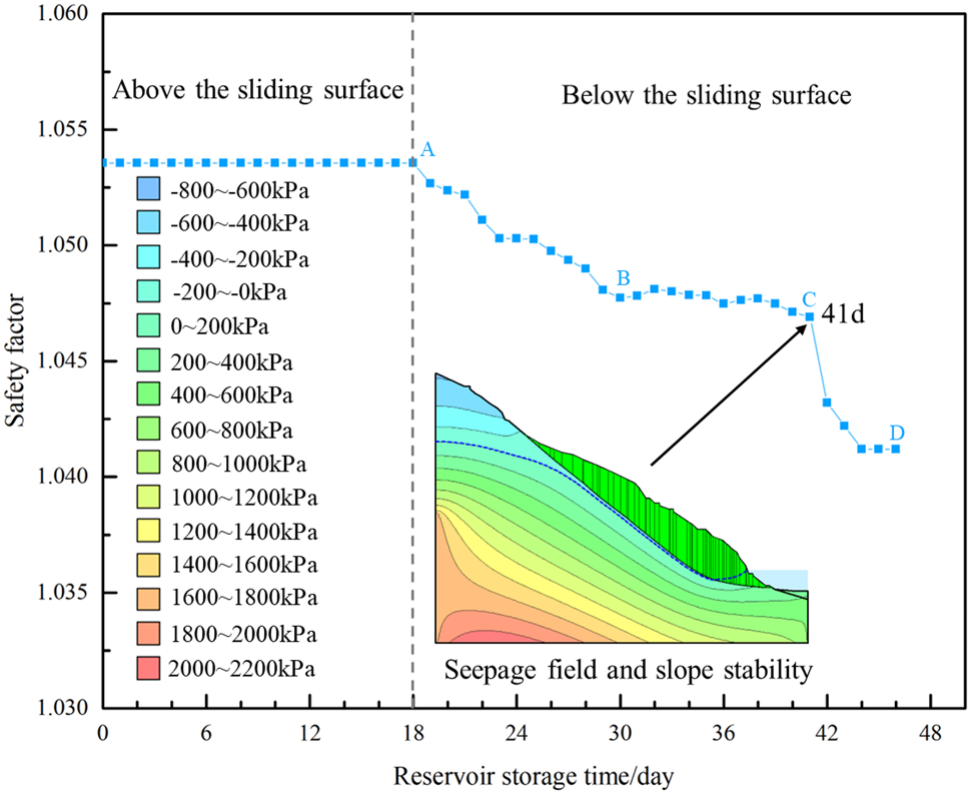
Safety factor variation curve.

Furthermore, according to the analysis of previous deformation and displacement monitoring data and seepage characteristics, this landslide exhibits a typical reservoir-induced delayed response with the relationship reservoir water level fluctuation rate (v) being greater than the permeability coefficient (c) of the sliding mass^10^. During the first 17 days of impoundment, as the reservoir water level was lower than the sliding surface, it had minimal impact on the stability of the landslide. However, starting from the 18th day, as the reservoir water level exceeded the sliding surface, the stability of the landslide began to decrease (Fig. 10 (A → B)). As the water level continued to rise, the decline in stability showed a slight slowdown (Fig. 10 (B → C)). When the reservoir water level reached its highest operational point, during the transition of groundwater seepage from a transient to a steady state, the buoyancy-induced reduction in weight further increased, leading to a continued reduction in the landslide's stability, reaching its lowest point (Fig. 10 (C → D)).

## Deformation mechanism

**Figure 11:**
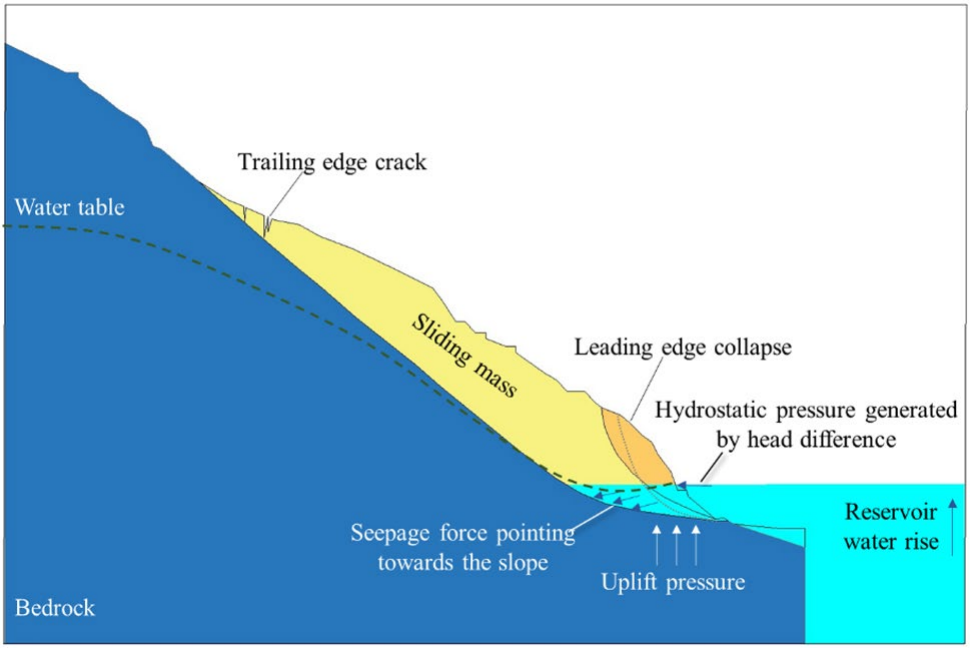
Deformation mechanism.

Based on a comprehensive assessment of the engineering geological conditions, deformation characteristics, displacement monitoring, and stability analysis of the Mala landslide, it is concluded that the condition of the leading-edge free surface is relatively favorable. As shown in Fig. 11, under the rising reservoir water level conditions, localized collapsing and sliding deformation occur in the front part of the landslide. This sliding at the front creates new frontal conditions, leading to stress redistribution within the slope and the gradual appearance of tension cracks at the rear of the slope. As the reservoir level continues to rise, the water level within the slope lags behind the reservoir water level, resulting in a water level difference. Water infiltrates into the slope, generating dynamic water infiltration pressure and pore static water pressure, with the buoyant force produced by pore static pressure being the dominant factor^10,11^. When the reservoir water level rises to 1401 m, on one hand, there is the application of static water pressure on the slope mass, leading to a reduction in the effective self-weight of the front part of the sliding mass^12^. On the other hand, due to the immersion effect of the reservoir water, the param of the anti-slide segment also decrease. Finally, the significant decrease in anti-slip force of the sliding mass leads to deformation and instability of the landslide.

## Conclusion

Based on field investigations and monitoring data, this paper provides a detailed account of the deformation characteristics of the Mala landslide. In conjunction with numerical simulation, it analyzes the changes in the seepage field and stability coefficients of the landslide as the reservoir water level rises. The main conclusions drawn are as follows:

1. The landslide as a whole exhibit a tendency to move in the downstream direction of the Lancang River, with the central part of the landslide located in the lower-central area of the sliding mass. Under current conditions, the Mala landslide is in a state of deep-seated creep deformation.
2. The Mala landslide is a typical buoyant force reduction type—retrogressive type landslide. During the period of impoundment, the landslide develops surface cracks sequentially from the front to the rear. After the reservoir water level stabilizes at its highest operational point, during the transition of groundwater seepage from a transient to a steady state, the buoyancy-induced reduction in weight intensifies the landslide deformation.
3. The deformation and instability of the Mala landslide exhibit a delayed response with the relationship reservoir water level fluctuation rate being greater than the permeability coefficient of the sliding mass. As the reservoir water level rises from low to high water levels, stability decreases. When the reservoir water level reaches its highest operational point, the landslide stability reaches its lowest point.

## Data Availability

The datasets generated and analysed during the current study are not publicly available due restrictions apply to the availability of these data but are available from the corresponding author on reasonable request.
